# *In vitro *susceptibility to quinine and microsatellite variations of the *Plasmodium falciparum *Na^+^/H^+ ^exchanger (*Pfnhe-1) *gene: the absence of association in clinical isolates from the Republic of Congo

**DOI:** 10.1186/1475-2875-10-37

**Published:** 2011-02-11

**Authors:** Sébastien Briolant, Stéphane Pelleau, Hervé Bogreau, Philippe Hovette, Agnès Zettor, Jacky Castello, Eric Baret, Rémy Amalvict, Christophe Rogier, Bruno Pradines

**Affiliations:** 1 Unité de Parasitologie, Unité Mixte de Recherche 6236, Institut de Recherche Biomédicale des Armées - Antenne de Marseille. Marseille, France; 2 Unité de Recherche en Pharmacologie et Physiopathologie Parasitaires, UMR MD3 Relation hôte-parasite, Pharmacologie et Thérapeutique, Institut de Recherche Biomédicale des Armées - Antenne de Marseille, Marseille, France; 3 Clinique Total, Division Médicale de Total Exploration et Production Congo Pointe-Noire, République du Congo

## Abstract

**Background:**

Quinine is still recommended as an effective therapy for severe cases of *Plasmodium falciparum *malaria, but the parasite has developed resistance to the drug in some cases. Investigations into the genetic basis for quinine resistance (QNR) suggest that QNR is complex and involves several genes, with either an additive or a pairwise effect. The results obtained when assessing one of these genes, the plasmodial Na^+^/H^+ ^exchanger, *Pfnhe-1*, were found to depend upon the geographic origin of the parasite strain. Most of the associations identified have been made in Asian strains; in contrast, in African strains, the influence of *Pfnhe *on QNR is not apparent. However, a recent study carried out in Kenya did show a significant association between a *Pfnhe *polymorphism and QNR. As genetic differences may exist across the African continent, more field data are needed to determine if this association exists in other African regions. In the present study, association between *Pfnhe *and QNR is investigated in a series of isolates from central Africa.

**Methods:**

The sequence analysis of the polymorphisms at the *Pfnhe-1 *ms4760 microsatellite and the evaluation of *in vitro *quinine susceptibility (by isotopic assay) were conducted in 74 *P. falciparum *isolates from the Republic of Congo.

**Results:**

Polymorphisms in the number of DNNND or NHNDNHNNDDD repeats in the *Pfnhe-1 *ms4760 microsatellite were not associated with quinine susceptibility.

**Conclusions:**

The polymorphism in the microsatellite ms4760 in *Pfnhe-1 *that cannot be used to monitor quinine response in the regions of the Republic of Congo, where the isolates came from. This finding suggests that there exists a genetic background associated with geographic area for the association that will prevent the use of *Pfnhe *as a molecular marker for QNR. The contribution of *Pfnhe *to the *in vitro *response to quinine remains to be assessed in other regions, including in countries with different levels of drug pressure.

## Background

Quinine (QN) remains a first-line drug for the treatment of severe malaria that is still used as a second-line therapy for uncomplicated malaria in many countries [1]. Despite the efficacy of QN against chloroquine-resistant *Plasmodium falciparum *isolates, reports of QN resistance (QNR) have been increasing. In the 1980s, the frequency of clinical failures increased in Southeast Asia and Africa [2-4]. The mechanism of QNR is complex, multigenic, and not well understood. QNR has been associated with mutations in both the *P. falciparum *multidrug resistance gene *mdr1 *(*Pfmdr1*) [5] and the chloroquine resistance transporter gene *Pfcrt *[6]. Other genetic polymorphisms, such as mutations in the resistance protein gene *Pfmrp *[7] and variations of microsatellite length in the sodium/hydrogen exchanger gene *Pfnhe-1 *[8], may contribute to QNR. Using quantitative trait loci (QTL) on the genetic cross of HB3 and Dd2 strains, Ferdig *et al *identified genes on chromosome 5, encoding *Pfmdr1*, on chromosome 7, encoding *Pfcrt *and on chromosome 13, encoding *Pfnhe-1*, which were associated with QN reduced susceptibility [8]. Sequences of Pfnhe-1 showed multiple and complex variations such as point polymorphisms at three separate codons (790, 894 and 950) and as microsatellite variations in three different repeat sequences (msR1, ms3580 and ms4760). However, the three point polymorphisms and microsatellite polymorphisms msR1 and ms3580 showed no significant association with QN susceptibility. The investigations of the microsatellite ms4760 polymorphisms in culture-adapted isolates from around the world showed that an increased number of the amino acid motif DNNND was associated with a decreased QN susceptibility, and that an increased number of NHNDNHNNDDD motifs was associated with an increased QN susceptibility [8,9]. The association of two repeats with a high QN inhibitory concentration of 50% (IC_50_) was found in a case of QN clinical failure in a traveller from Senegal [10]. In contrast, a recent multivariate analysis performed on 83 freshly collected clinical isolates from Madagascar and 13 African countries did not find an association between QN susceptibility and *Pfnhe-1 *microsatellite polymorphisms [11]. A similar absence of association was observed in 91 isolates from various countries on different continents (Pelleau S. *et al*. submitted). Given that the influence of *Pfnhe *on QN susceptibility has been shown to be strain-dependent, these apparently conflicting results may be explained, in part, by differences in the geographic origin of the parasites analysed, as their local selection history and genetic background varies.

Thus, further epidemiological investigation is required to determine the context in which *Pfnhe *can be used as a molecular marker of QNR. In this work, ms4760 polymorphism was analized and its association tested with *in vitro *susceptibility to QN in African isolates from a single geographical region, Pointe-Noire in the Republic of Congo.

## Methods

### Reference culture-adapted strains and clinical isolates of *Plasmodium falciparum*

Between March 2005 and January 2006, 74 *P. falciparum *clinical isolates were collected from patients with uncomplicated malaria at the Medical Service of Total Exploration et Production, Pointe-Noire (Republic of Congo) [12]. Two cloned strains of *P. falciparum *were used for quality control (3D7 Africa and W2 Indochina). These clones were obtained from MR4-ATCC (Manassas, VA, USA). They were maintained in culture in RPMI 1640 (Invitrogen, Paisley, UK) that was supplemented with 10% human serum (Abcys S.A. Paris, France) and buffered with 25 mM HEPES and 25 mM NaHCO_3_. The parasites were grown in type A+ human blood under controlled atmospheric conditions (10% O_2_, 5% CO_2_, and 85% N_2_) at 37°C with a humidity of 95%. They were synchronized twice with sorbitol before use [13]. Clonality was verified using PCR genotyping of the polymorphic genetic markers, *msp1 *and *msp2*, and the microsatellite loci [14,15].

### *In vitro *assay

The QN was purchased from Sigma (Saint Louis, MO, USA). The QN was first dissolved in methanol and then diluted in water to obtain final concentrations ranging from 5 to 3200 nM.

For the *in vitro *isotopic microtest, 200 μl of the suspension of synchronous parasitized red blood cells (final parasitaemia, 0.5%; final haematocrit, 1.5%) per well were plated in 96-well plates that contained serial QN concentrations. Parasite growth was assessed by adding 1 μCi of tritiated hypoxanthine with a specific activity of 14.1 Ci/mmol (Perkin-Elmer, Courtaboeuf, France) to each well at time zero. The plates were then incubated for 48 h under controlled atmospheric conditions. Immediately after incubation, the plates were frozen and then thawed to lyse the erythrocytes. The contents of each well were collected on standard filter microplates (Unifilter GF/B; Perkin-Elmer) and washed using a cell harvester (Filter-Mate Cell Harvester; Perkin-Elmer). The filter microplates were dried and 25 μl of a scintillation cocktail (Microscint O; Perkin-Elmer) was added to each well. The radioactivity incorporated by the parasites was then measured using a scintillation counter (Top Count; Perkin-Elmer). The drug concentration that could inhibit 50% of the parasite growth (IC_50_) was calculated assuming that it corresponded to the drug concentration at which the incorporation of tritiated hypoxanthine by the parasite was 50% of that incorporated in the drug-free, control wells. IC_50 _values were determined using a non-linear regression analysis of log-based dose-response curves (Riasmart, Packard, Meriden, USA).

### Genotyping of the *Pfnhe *ms4760 microsatellite polymorphisms

Parasite DNA from 100 μl of infected blood was extracted using the E.Z.N.A. Blood DNA kit (Omega Bio-Tek, GA, USA). A sequence containing the previously described ms4760 microsatellite [8] was amplified using the *pfnhe*-3802F 5'-TTATTAAATGAATATAAAGA-3' and *pfnhe*-4322R 5'-TTTTTTATCATTACTAAAGA-3' primers. Sequencing was performed using ABI Prism Big Dye Terminator v1.1 Cycle Sequencing Ready Reaction Kits (Applied Biosystems, CA, USA), as directed by the manufacturer. Sequences were analysed with BioEdit sequence alignment editor (version 7.0.9.0) software.

### Statistical analysis

Data were analysed using R software (version 2.10.1) and GraphPad Prism (version 5.01). Differences between the median QN IC_50 _values of isolates harbouring one, two or three DNNND repeats were tested using the Kruskal-Wallis test. The median QN IC_50 _values of isolates with one or two NHNDNHNNDDD repeats were compared using the Mann Whitney test.

## Results

**Figure 1:**
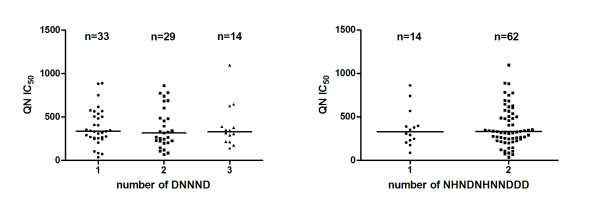
**Distribution of quinine (QN) IC_50 _versus the repeats number of DNNND or NHNDNHNNDDD**. The horizontal bars indicate median values.

The mean QN IC_50 _of the 74 isolates was 381.9 nM, CI95% [330.7-433.1] (min-max: 36-1097 nM). Four isolates (5.4%) had a QN IC_50 _> 800 nM (Table 1) that corresponds to the *in vitro *QNR threshold. Twenty-seven different *Pfnhe-1 *genotypes were observed among the 74 isolates, and eighteen were identified as new genotypes (GenBank accession numbers FJ392810 to FJ392827). The DNNND repeats varied from 1 to 3. There was no statistically significant association (p = 0.84) between the median QN IC_50 _value and the number of DNNND repeats (medians at 335, 316 and 330 nM, for isolates harbouring one, two or three repeats, respectively) (Figure 1). No statistically significant association was observed (p = 0.92) between the median QN IC_50 _value of isolates and the number of NHNDNHNNDDD repeats (medians at 328 and 329 nM, for isolates with one and two repeats, respectively).

**Table 1:** *In vitro *susceptibility and *Pfnhe*-1 polymorphisms of the 74 *Plasmodium falciparum *isolates from the Republic of Congo

Isolates	Cpm without drug	Cpm With highest QN concentration	QN IC_50_	No DNNND repeat	No NHNDNHNNDDD repeat	No genotype profile
16517	23404	1440	1097	3	2	ms4760-27
16612	49650	8273	888	1	2	ms4760-3
16520	6512	1077	882	1	2	ms4760-3
16094	2007	620	862	2	1	ms4760-24
16980	2151	980	782	2	2	ms4760-33
17267	2558	455	776	2	2	ms4760-1
16631	1807	282	750	1	2	ms4760-3
16698	1263	353	740	2	1	ms4760-6
16960	2011	714	689	2	2	ms4760-1
16958	19732	861	645	3	2	ms4760-30
16983	3710	943	627	3	2	ms4760-1
16957	19773	1300	615	1	2	ms4760-29
16966	2264	545	604	2	2	ms4760-31
16941	18540	1175	576	1	2	ms4760-3
16991	2804	815	567	1	1	ms4760-36
16953	5675	1249	564	1	2	ms4760-12
16539	44532	4112	537	1	2	ms4760-3
17250	6558	606	506	1	2	ms4760-3
16942	10927	1163	502	1	2	ms4760-3
16959	9886	914	488	2	2	ms4760-1
17008	2151	961	482	1	2	ms4760-35
16986	2302	624	481	2	2	ms4760-1
16968	8477	962	457	2	2	ms4760-1
17249	1276	412	408	1	2	ms4760-22
17216	3179	539	404	1	2	ms4760-38
17253	9717	1091	396	2	1	ms4760-6
16987	7232	938	388	3	1	ms4760-15
16349	28606	2048	377	3	1	ms4760-25
17231	3545	389	354	1	2	ms4760-39
16518	8912	1361	351	3	1	ms4760-7
16955	9379	1188	346	3	2	ms4760-27
17211	6451	655	346	1	2	ms4760-3
16695	27421	2316	341	1	2	ms4760-3
16996	1347	597	340	2	2	ms4760-34
17208	18356	1208	335	1	2	ms4760-3
16536	6617	847	333	2	2	ms4760-1
16365	4631	724	332	1	2	ms4760-22
16979	7449	990	326	2	2	ms4760-18
17202	11053	929	319	1	2	ms4760-3
16364	12210	1990	316	2	2	ms4760-26
16331	8912	1113	314	3	2	ms4760-9
15868	1530	498	309	1	2	ms4760-22
16610	4063	961	305	3	1	ms4760-7
16535	16007	1274	291	3	1	ms4760-15
16323	43074	5859	290	1	2	ms4760-12
16992	9464	1111	276	1	2	ms4760-12
17237	12385	873	269	1	2	ms4760-3
17305	21667	1398	269	2	2	ms4760-3
16305	11269	917	265	1	2	ms4760-3
17303	18553	1646	264	2	2	ms4760-1
17212	2755	520	259	1	2	ms4760-3
16304	16194	738	254	2	2	ms4760-1
17241	5758	447	254	1	2	ms4760-3
16970	2359	705	247	1	1	ms4760-32
16995	7642	1004	247	2	2	ms4760-1
17210	11018	1370	244	1	2	ms4760-22
17233	12100	956	231	2	1	ms4760-6
17265	1593	238	226	2	2	ms4760-1
17214	2421	316	224	2	2	ms4760-37
17209	3499	680	216	3	2	ms4760-9
17007	3633	1159	212	3	2	ms4760-9
16116	11417	999	204	2	2	ms4760-18
17201	25594	2530	204	1	1	ms4760-36
17304	24453	1982	199	2	2	ms4760-1
16086	10918	699	174	3	1	ms4760-15
17283	4391	315	146	3	2	ms4760-1
17010	2897	1103	141	3	2	ms4760-9
16117	17474	1291	121	2	2	ms4760-1
16078	1654	396	106	2	2	ms4760-1
16085	5229	707	86	2	1	ms4760-6
17301	39337	2016	84	1	2	ms4760-12
15867	6893	739	73	1	2	ms4760-3
16091	17320	1695	67	2	2	ms4760-1
16077	4539	353	36	1	2	ms4760-23

Cpm = count per minute

IC_50 _= Inhibitory concentration 50% in nM

QN = quinine

*Pfnhe- 1 *ms4760 microsatellite genotype profiles according to the GenBank

## Discussion

QN has been used to treat malaria for more than 350 years in Africa, with little emergence and spread of resistance. QN remains the first line anti-malarial drug for the treatment of complicated malaria in Europe and Africa. However, despite the efficacy of QN against chloroquine-resistant strains, the emergence of QN resistance has been observed. The first cases of QN clinical failure were observed in Brazil and Asia in the 1960s; then in the 1980s, clinical failures became more frequent in Southeast Asia, South America and Africa. However, QN resistance is not yet a significant problem in Africa, and QN remains the first-line drug to treat severe malaria and a second-line therapy for uncomplicated malaria in some areas of Africa.

Although some reports of QN treatment failure exist, it is difficult to confirm QN resistance because of the short elimination half-life of the drug, the requirement to administer it three times a day for at least five days, drug intolerance that often leads to poor compliance and a lack of reliable data on the correlation between QN IC_50 _and clinical failure. Maximizing the efficacy and longevity of QN as a tool for the control of malaria will depend critically on the pursuit of intensive research toward the identification of *in vitro *markers of QNR and the implementation of *in vitro *and *in vivo *surveillance programs, such as those championed by the Worldwide Antimalarial Resistance Network [16,17]. Specifically, there is a need to identify molecular markers that effectively predict QN resistance and enable the active surveillance of temporal trends in parasite susceptibility [18]. The present study aimed to test the association between the *Pfnhe *polymorphism and QN susceptibility in clinical isolates from Pointe-Noire in Congo to assess the validity of *Pfnhe *as a molecular marker of QN susceptibility in this region.

The level of susceptibility to QN remained high, with a mean QN IC_50 _of 382 nM and with 4/74 isolates (5.4%) that exceed the QNR threshold of 800 nM [19]. The threshold at which QN *in vitro *susceptibility is considered to be compromised is arbitrary, and different studies have set the following thresholds: 300 nM [20], 500 nM [21] or 800 nM. If a QNR threshold of 500 nM is applied here, 19/74 isolates exceed this value. These results suggest that QN treatment is still effective against the chloroquine-resistant parasites that are found in this area, where the prevalence of chloroquine-resistant parasites has reached 75% [12].

A high level of *Pfnhe-1 *microsatellite sequence polymorphisms was found (27 genotypes for 74 isolates). Previous studies reported from 8 genotypes for 71 isolates [8] to 35 for 60 isolates [22]. DNNND and NHNDNHNNDDD repeat numbers ranged from 1 to 3 and 1 to 2, respectively. However, neither DNNND nor NHNDNHNNDDD repeat polymorphisms were linked to QN susceptibility.

According to this study and previous studies, the association between QN *in vitro *susceptibility and the *Pfnhe-1 *microsatellite genotype appears to be geographically dependent.

One explanation for this could be variation in genetic background. A specific genetic background observed in Asia may allow the observed contribution of *Pfnhe *polymorphism on QN *in vitro *susceptibility. This explanation is consistent with: i) the first evidence of *Pfnhe*-QNR association from QTL analysis using Americano-Asian cross strains [8], ii) that most associations identified have been shown among Asian strains [8,9,22] and iii) that at least 5 genes spanning the *P. falciparum *genome influence the QNR *in vitro *phenotype with an additive effect or with pairwise interactions [8].

Additionally, these genetic dissimilarities between African and Asian plasmodial populations may be accentuated by different local selection histories. The best-documented genomic modifications by a local selection process relate to drug pressure. Several studies have shown that drug pressure may involve extended linkage disequilibrium around a drug resistance associated gene [23]. This is characterized by a strong genetic diversity loss, called a selective sweep. This process stretch may be modified by: i) drug use and ii) malaria transmission level. A reduced drug use and higher malaria transmission level in Africa would be consistent with the lower selective sweep and an absence of linkage disequilibrium between *Pfnhe *and other cooperative drug response genes or selected compensatory mutations. For example, QTL analysis on chromosome 13 located 60 genes of unknown function as being close to *Pfnhe *[8]. They would be more or less linked depending on drug pressure and malaria transmission level if QN pressure selects at least one of them. In Kenya there is an association between two DNNND repeats in the ms4760 *Pfnhe *microsatellite and a reduced susceptibility to quinine in 29 *P. falciparum *isolates [24]. This is consistent with the historic precedent of the spread of drug resistance around the world. The emergence of chloroquine resistance in Asia was followed by an initial introduction into East Africa and spreading across the African continent. Geographic proximity may explain plasmodial population migration. Moreover, East African plasmodial populations may exhibit genetic dissimilarities to other African populations [14].

As the QN response is controlled by multiple genes with complex interactions, one can expect: i) higher sensitivity to genetic background than if the response was controlled by only one gene, ii) higher sensitivity to parameters that may cause linkage disequilibrium between genes and iii) various combinations of gene polymorphisms may result in similar QNR phenotypes.

## Conclusions

In summary, although studies have demonstrated that *Pfnhe-1 *contributes to QNR [24], the present study and another recent study [11] did not show an association between *Pfnhe-1 *polymorphism and QNR. Currently, *Pfnhe-1 *cannot be used as a QNR molecular marker when evaluating field isolates, especially in Africa in the context of a low level of quinine selection pressure. Further studies, using more parasite samples from South East Asia with reduced susceptibility to QN, are required to confirm or reject the use of the *Pfnhe-1 *gene as a QNR marker in this geographic region.

## Conflict of interests

The authors declare that they have no competing interests.

## Authors' contributions

SB, SP, CR and BP conceived and designed the experiments. SB, AZ, EB and RA performed the experiments. SB, SP, HB, and BP analysed the data. PH, AZ and JC contributed reagents, materials and analytical tools. SB, SP, HB, PH, CR and BP wrote the paper. All the authors read and approved the final manuscript.
